# Mode of delivery and preterm birth in subsequent births: A systematic review and meta-analysis

**DOI:** 10.1371/journal.pone.0213784

**Published:** 2019-03-14

**Authors:** Yinghui Zhang, Jie Zhou, Yubo Ma, Li Liu, Qing Xia, Dazhi Fan, Wen Ai

**Affiliations:** 1 Department of Obstetrics and Gynecology, Foshan Chancheng Central Hospital, Foshan, Guangdong, China; 2 Department of Pediatrics, Foshan Chancheng Central Hospital, Foshan, Guangdong, China; 3 Department of Epidemiology and Biostatistics, School of Public Health, Anhui Medical University, Hefei, Anhui, China; 4 Department of Library, the First Affiliated Hospital, College of Medicine, Zhejiang University, Hangzhou, Zhejiang, China; 5 Menzies Institute for Medical Research, University of Tasmania, Hobart, Tasmania Australia; 6 Foshan Institute of Fetal Medicine, Southern Medical University Affiliated Maternal & Child Health Hospital of Foshan, Foshan, Guangdong, China; University of South Florida, UNITED STATES

## Abstract

Preterm birth continues to be an important problem in modern obstetrics and a large public health concern and is related to increased risk for neonatal morbidity and mortality. The aim of this study was to evaluate the data in the literature to determine the relationships between mode of delivery (cesarean section and vaginal birth) in the first pregnancy and the risk of subsequent preterm birth from a multi-year population based cohorts (PROSPERO registration number: 42018090788). Five electronic databases were searched. Observational studies that provided mode of delivery and subsequent preterm birth were eligible. Ten cohort studies, involving 10333501 women, were included in this study. Compared with vaginal delivery, women delivering by previous cesarean section had a significantly higher risk of preterm birth in subsequent births (RR 1.10, 95%CI 1.01–1.20). After adjusting confounding factors, there was still statistical significance (aRR 1.12, 95%CI 1.01–1.24). However, both before and after adjustment, there was no difference among very preterm birth (RR 1.14, 95%CI 0.90–1.43; aRR 1.16, 95%CI 0.80–1.68; respectively). To the best of our knowledge, this is the first systematic review and meta-analysis that suggests previous cesarean section could increase the risk of preterm birth in subsequent pregnancies. The result could provide policy makers, clinicians, and expectant parents to reduce the occurrence of unnecessary cesarean section.

## Introduction

Preterm birth, defined as iatrogenic or spontaneous delivery before 37 completed gestational weeks, continues to be an important problem in modern obstetrics and remains a large public health concern. It affects 7.2% and 9.6% of pregnancies in China and United States, respectively [1, 2], and about 15 million pregnancies worldwide each year [3]. It is related to increased risk for neonatal morbidity and mortality [4, 5]. Most convincing are the findings that preterm birth is significantly associated with various adverse health outcomes, including low birth weight, visual and hearing impairment, neurodevelopmental impairment, lung disease, neonatal and infant death, and maternal adverse cardiovascular outcomes [6–9]. It is still a global priority to prevent preterm birth, although the exact pathogenesis is poorly understood [10]. Cesarean delivery rates varied markedly across worldwide and data showed that it increased in most countries during the past decade [11]. In China, it reported that 32.7% of births were delivered by cesarean between 2008 and 2014 [12]. Several investigations have demonstrated that previous cesarean delivery can elevate the risk of maternal complications including bleeding and intrauterine infection. More importantly, it can also increase the risk of adverse reproductive outcomes including ectopic pregnancy, uterine rupture, morbidly adherent placenta, hysterectomy, and preterm birth in subsequent pregnancies [13–15].

Over the past two decades, several cohort studies have investigated the association between previous mode of delivery and preterm birth in subsequent births. The positive association between cesarean section in the first pregnancy and subsequent preterm birth has been well documented [16–19]. However, other cohort studies [20, 21] showed that there didn’t seem to be enough evidence to come to a conclusion on the association between cesarean delivery in the first pregnancy and preterm birth in subsequent pregnancies. After adjusting for confounder factors, researchers [22, 23] even found that previous cesarean delivery could reduce the incidence of subsequent preterm birth. It is unclear whether previous cesarean section could increase the risk of subsequent preterm birth and to what extent compared with previous vaginal birth. Small cohort studies may be underpowered to distinguish the risk of previous cesarean section in subsequent pregnancies. Therefore, it is required a comprehensive study to clarify the risk of previous cesarean delivery in the subsequent pregnancies.

Through a systematic review and meta-analysis, our aim of this study was to evaluate the data in the literature to determine the relationships between mode of delivery (cesarean section and vaginal birth) in the first pregnancy and the risk of subsequent preterm birth from a multi-year population based retrospective cohort. Understanding the intertwined relationship between mode of delivery (cesarean section and vaginal birth) in the first pregnancy and the risk of subsequent preterm birth may remind policy makers, gynecologists and obstetricians, and expectant parents to reduce the occurrence of unnecessary cesarean section.

## Materials and methods

We reported this systematic review and meta-analysis following the Preferred Reporting Item for Systematic Reviews and Meta-analyses statement [24]. Before data collecting, it was prospectively registered with the University of York Centre for Reviews and Dissemination International Prospective Register of Systematic Reviews (PROSPERO Identifier: CRD42018090788). It was designed a priori to define methods for searching terms, assessing the quality of included studies, collecting, extracting, and analyzing data in the review protocol.

### Search strategy

In this systematic review and meta-analysis, published articles were searched with no language restrictions by two independent reviewers (LL and YM) in PubMed, Web of Science, Embase, Elsevier ScienceDirect and the Cochrane Library (updated on November 27, 2018). The search terms were “mode of delivery”, “cesarean delivery”, “cesarean section” “vaginal delivery”, “preterm birth”, and “preterm delivery”. A detailed search processes used for the PubMed was shown in S1 Text. Bibliographies of identified articles were also reviewed and searched manually for additional references.

### Study selection

Observational studies (including cohort and case-control) assessing previous mode of delivery (cesarean delivery vs. vaginal delivery) and preterm birth in subsequent births were included. Only single pregnancy in our analyses was included, and twin pregnancy was excluded. First, based on the predetermined eligibility criteria, titles and abstracts of the potentially eligible articles were independently screened by YM and LL. Any duplicates were excluded. Then, potentially eligible studies were assessed and appraised full texts. Two authors (YM and LL) independently read the full text of the included studies and extracted the relevant data via a recognized data extraction form in Microsoft Excel software. Study characteristics, such as published journal and year, and first author’s name, study setting, such as period of enrollment and country, study design, study population characteristics, such as number of participants, gestational week, and outcomes such as risk estimates with corresponding confidence intervals, and confounding factors were extracted in each included study. Diagnosis and confirmation of preterm birth (before gestational week 37) and very preterm birth (before gestational week 32) were done according to the criteria of each study. If there were disagreement or uncertainty, consensus with the team members was used to resolve it.

### Study quality assessment

To assess the risk of bias of observational studies, two authors (LL and QX) used the Newcastle Ottawa Scale (NOS) [25, 26] to assess it. Individual quality items were assessed using stars including selection, comparability, and outcome. The selection included four items. Each item was one star. The comparability included one item, and the item can gain two stars. The outcome included three items, and each item was also one star. Each study was got the number of stars and the maximum number of star was nine in one study. It was considered high quality if the studies gained six or more stars [27]. Consensus with the team members was used to resolve it if there were disagreement or uncertainty.

### Statistical analysis

Relative risk (RR) with 95% confidence intervals (CI) was collected from the included articles. To examine the statistical heterogeneity, Higgins *I*^*2*^ statistics was used. According to *I*^*2*^-value, the statistical heterogeneity was divided into three categories: mild (< 25%), moderate (25–50%), and large (> 50%) [28, 29]. Meanwhile, Cochran’s Q statistic was also applied. Based on the heterogeneity, random- or fixed-effects meta-analysis was used to calculate the pooled effect value [30, 31]. To examine potential publication bias, Begg’s and Egger’s test were used. To assess whether study influenced the overall results, sensitivity analysis was performed. Two-sided *P*-value of 0.05 was considered statistically significant. Data analysis was completed using Stata 12.0 (Stata Corporation, College Station, TX, USA).

## Results

### Characteristics of included studies

**Fig 1:**
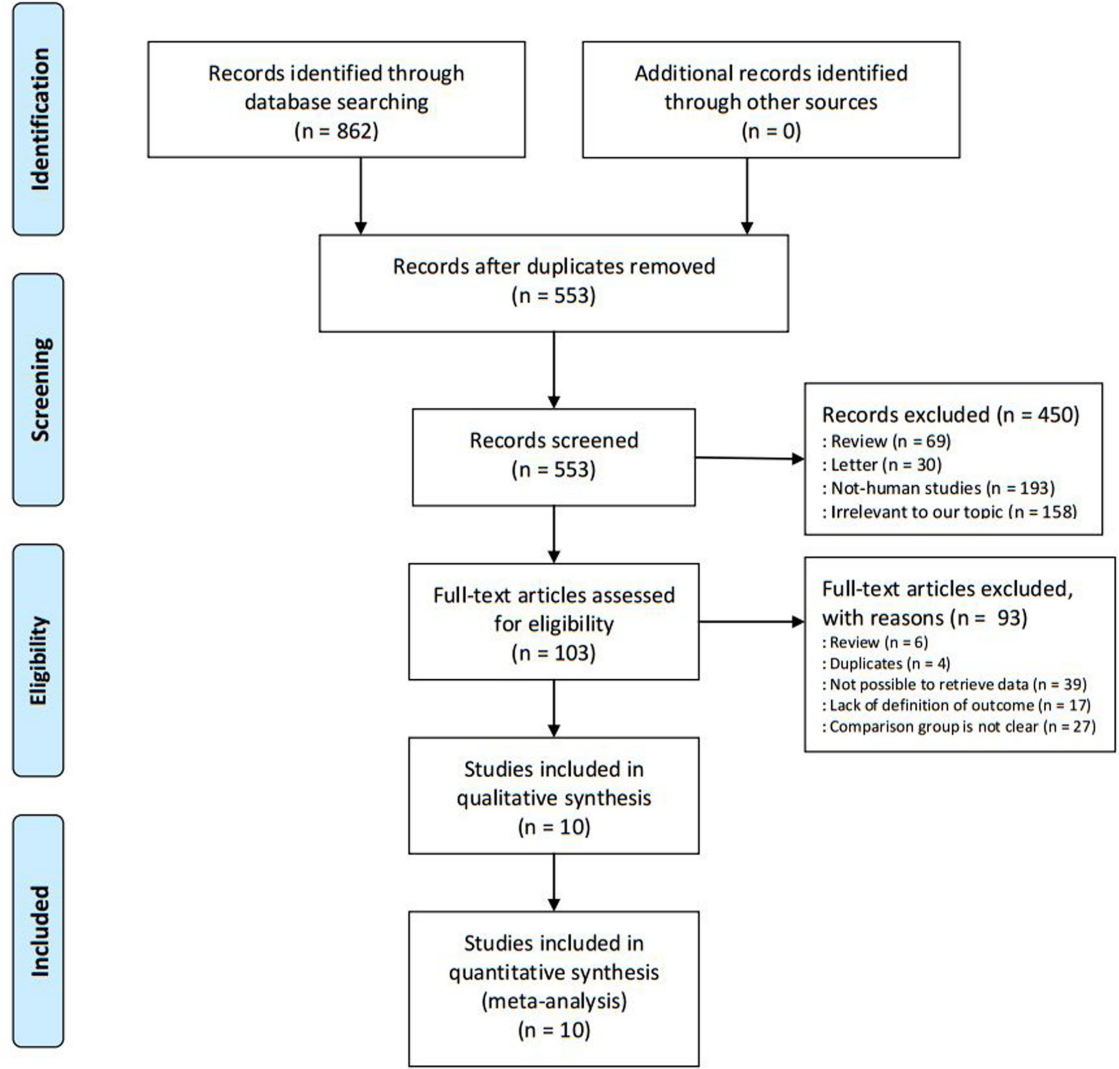
Flow chart for search and selection of studies for inclusion in this meta-analysis.

The flow diagram, detailed process of inclusion and exclusion criteria (PRISMA template), was shown in Fig 1. Eight hundred and sixty-two unique citations were identified with the initial search. Six hundred and fifty-nine relevant citations were excluded after careful review the titles and abstracts. One hundred and three were selected for full-text review, and ninety-three of these were excluded, leaving 10 retrospective cohort studies [16, 17, 19–23, 32–34]. Table 1 presented the detailed characteristics and outcomes of these studies. The sample size ranged from 31573 to 8772705, and year of publication dated from 2001 to 2018. Total 10333501 women were included in these studies, and the women in previous cesarean section and vaginal delivery group were 2019506 and 8313995, respectively. Reported preterm births was 8506349 (rang from 27556 to 7297132). Four included studies [16, 19, 23, 32] reported the very preterm births. Eight of ten studies also reported the adjusted results [16, 17, 21–23, 32–34]. According to the Newcastle-Ottawa Scale, a total of 8.8 points were awarded for the ten included studies. Table 2 showed the detailed score of each article.

**Table 1:** Characteristics of the studies included in the meta-analysis.

Author, Year	City/Country	During	Study Design	Follow-up (year)	Sample Size (CS group/VD group)	Adjusted Confounding Factors	NOS Score
Yasseen Iii AS, 2018	Ontario/Canada	2005–2012	Retrospective cohort	9.5	481531 (119983/361548)	Adjusted for maternal age at birth, year and quarter of infant birth date, socioeconomic position measured using the material and social deprivation index, number of previous pregnancies, smoking during pregnancy, a history of preterm birth, and pre-existing diabetes and/or hypertension	9
Jackson S, 2012	/Denmark	1994–2010	Retrospective cohort	10	31573 (4030/27543)	Controlled for age, body mass index, tobacco, alcohol, socioeconomic status	9
Salihu HM, 2011	Missouri/USA	1978–2005	Retrospective cohort	19.5	450141 (146443/303698)	Adjustment for infant sex, maternal age, race, BMI, educational level, marital status, smoking and alcohol use during pregnancy, inter-pregnancy interval, adequacy of prenatal care and history of SGA or LGA, respectively	9
Huang X, 2011	/USA	1995–2002	Retrospective cohort	12.5	8772705 (1638456/7134249)	Adjusting variables: mother age, race, education years, prenatal care in first trimester, marital status, child sex.	9
Wood SL, 2008	Alberta/Canada	1991–2004	Retrospective cohort	10.5	157929 (30110/127819)	Unadjusted	8
Kennare R, 2007	South Australian/Australia	1998–2003	Retrospective cohort	6.5	36038 (8725/27313)	Adjusted for age, indigenous status, patient type, smoking, pregnancy interval, hypertension, diabetes, antepartum hemorrhage, history of termination of pregnancy	9
Taylor LK, 2005	New South Wales/Australia	1998–2003	Retrospective cohort	4.5	136101 (25596/110505)	Adjusted for maternal age; prior uterine curettage; smoking in pregnancy; health insurance status (public/private); ethnicity (Australian born non-Indigenous, Australian Indigenous, non-Australian born); socio-economic group; pre-existing diabetes; gestational diabetes; pre-existing hypertension; pregnancy-induced hypertension and infant sex.	9
Hemminki E, 2005	/Finland	1987–1998	Retrospective cohort	12.5	51220 (8534/42686)	Adjusted for age, smoking, and infant sex at second birth	9
Smith GC, 2003	Scotland/UK	1980–1998	Retrospective cohort	14	120633 (17754/102879)	Adjusted for maternal age, height, social deprivation quintile, and smoking status	9
Lydon-Rochelle M, 2001	Washington/USA	1987–1996	Retrospective cohort	9.5	95630 (19875/75755)	Unadjusted	8

CS: Cesarean Section; NOS: Newcastle-Ottawa Scale; VD: Vaginal Delivery

**Table 2:** Quality assessment of included cohort studies using the Newcastle-Ottawa Scale.

Cohort studies Author (year)	Selection				Comparability		Outcome			Total quality scores
	Representativeness of the exposed cohort	Selection of the non-exposed cohort	Ascertainment of exposure	Incident events			Assessment of outcome	Length of Follow-up	Adequacy of Follow-up of cohort	
Yasseen Iii AS, 2018	★	★	★	★	★	★	★	★	★	9
Jackson S, 2012	★	★	★	★	★	★	★	★	★	9
Salihu HM, 2011	★	★	★	★	★	★	★	★	★	9
Huang X, 2011	★	★	★	★	★	★	★	★	★	9
Wood SL, 2008	★	★	★	★	★		★	★	★	8
Kennare R, 2007	★	★	★	★	★	★	★	★	★	9
Taylor LK, 2005	★	★	★	★	★	★	★	★	★	9
Hemminki E, 2005	★	★	★	★	★	★	★	★	★	9
Smith GC, 2003	★	★	★	★	★	★	★	★	★	9
Lydon-Rochelle M, 2001	★	★	★	★	★		★	★	★	8

### Meta-analysis

**Fig 2:**
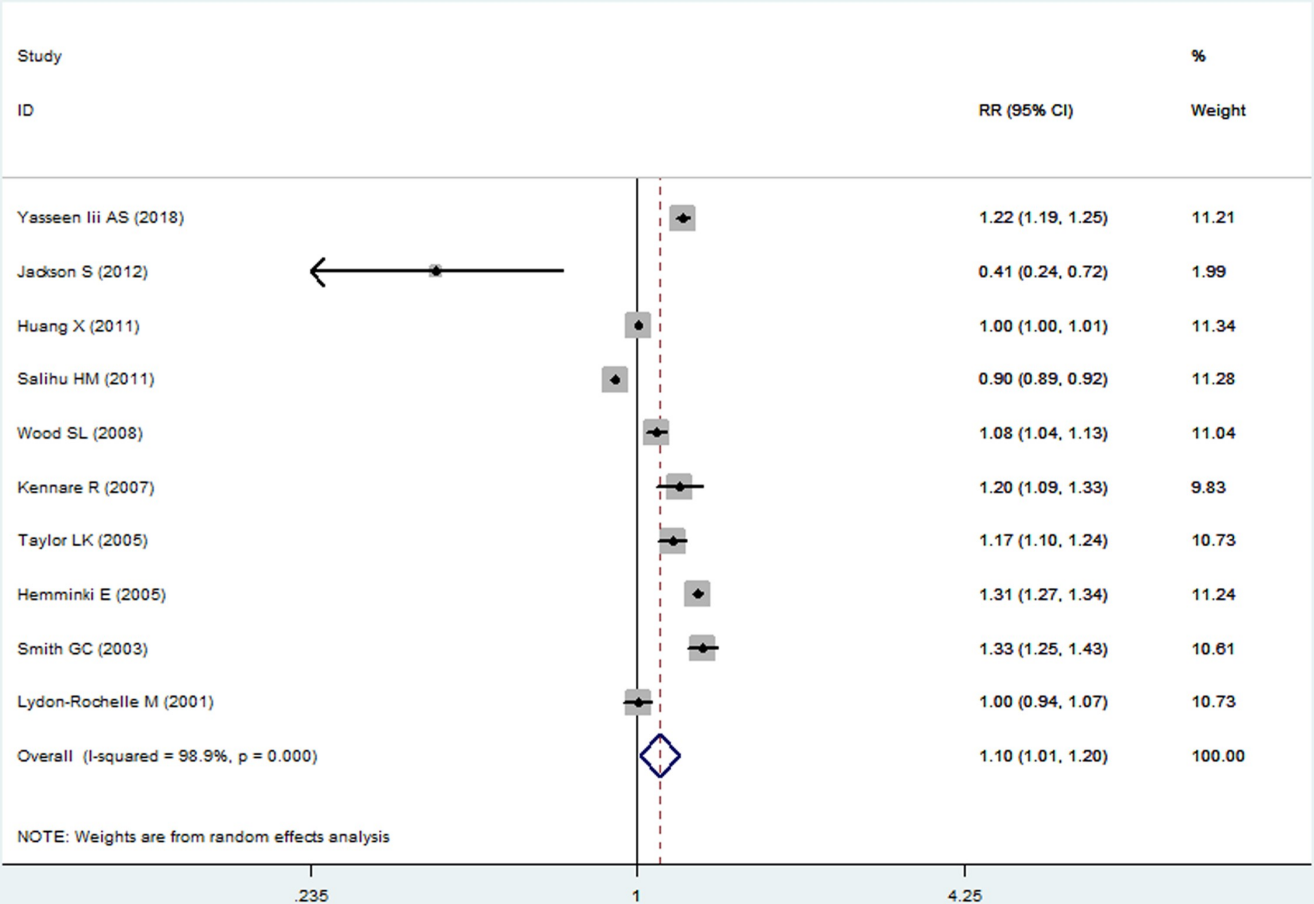
Pooled risk estimates of mode of delivery for preterm birth in subsequent births (cesarean section vs. vaginal delivery).

In the quantitative meta-analysis, ten retrospective cohort studies, involving 10333501 women, were included. Compared with vaginal group, women delivering by cesarean section in the last pregnancy had a significantly higher risk of preterm birth in subsequent births (RR 1.10, 95%CI 1.01–1.20, *I*^*2*^ = 98.8%; Fig 2). *I*^*2*^-value indicated that there was a high heterogeneity after pooling together. However, Begg’s and Egger’s tests showed there was no small-study effects (z = 0.09, p = 0.929; t = 1.50, p = 0.172) in the publication bias. Sensitivity analysis showed each single study did not influence the stability of the overall result.

**Fig 3:**
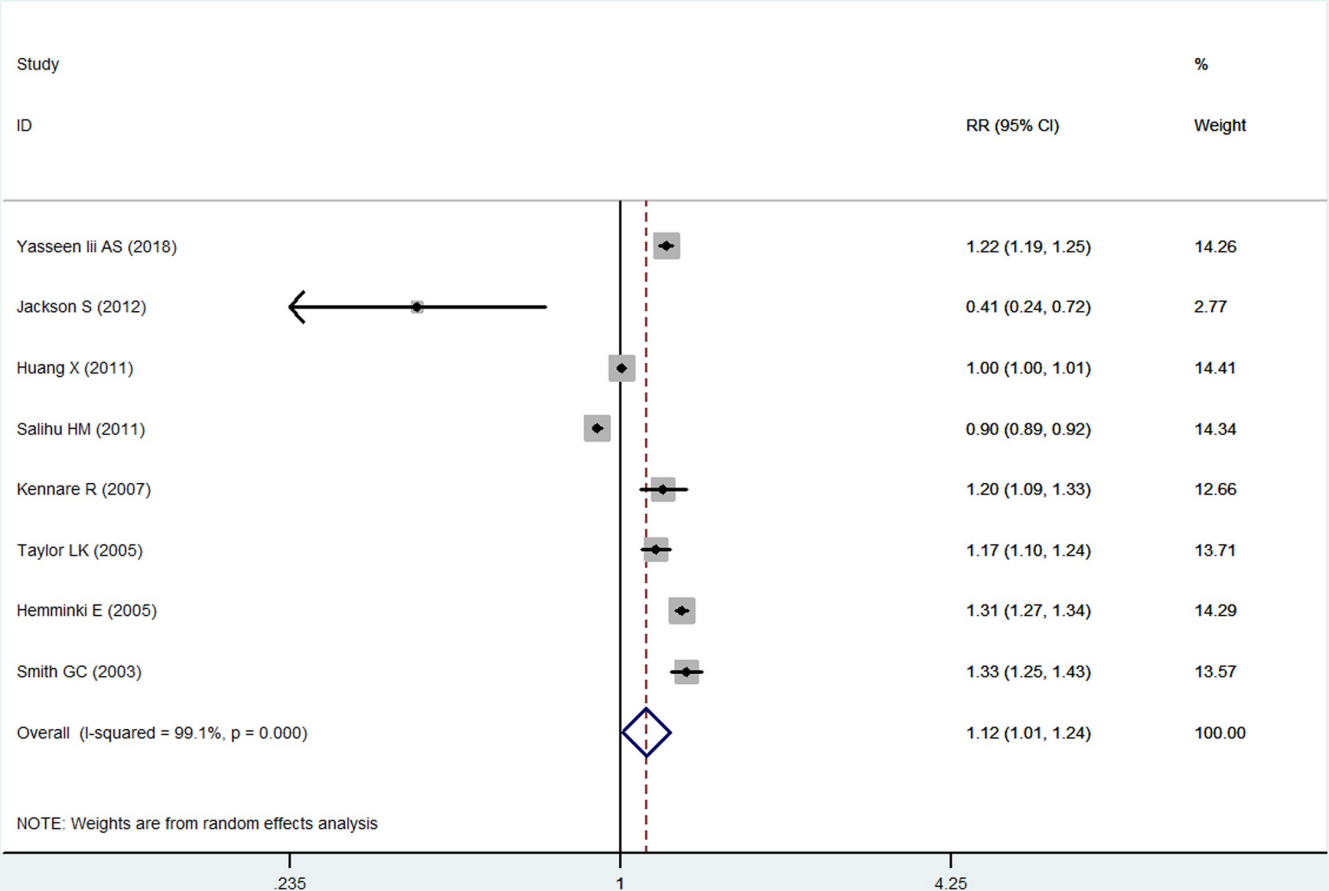
Pooled adjusted risk estimates of mode of delivery for preterm birth in subsequent births (cesarean section vs. vaginal delivery).

Eight studies, including 10079942 women, reported the adjusted results. Increased risk of preterm birth was also found in subsequent births for women delivering by cesarean section in the last pregnancy (aRR 1.12, 95%CI 1.01–1.24, *I*^*2*^ = 99.1%; Fig 3). Begg’s and Egger’s tests also showed no significant publication bias in the adjusted results (z = -0.12, p = 0.999; t = 1.42, p = 0.205). Similar results indicated that the result was also stable.

Four studies, including 764741 women, involved very preterm birth. However, both before and after adjustment, there were no found to be statistically significant in the risk of subsequent very preterm birth for women who had delivered by cesarean section in the last pregnancy (RR 1.14, 95%CI 0.90–1.43, *I*^*2*^ = 93.8%; aRR 1.16, 95%CI 0.80–1.68, *I*^*2*^ = 95.0%; respectively). There were no existing publication bias before and after adjustment was performed (z = 1.02, p = 0.308; t = 3.56, p = 0.071 and z = 0.001, p = 0.999; t = 2.39, p = 0.252, respectively) using Begg’s and Egger’s tests.

## Discussion

This is the first systematic review and meta-analysis, to our knowledge, that utilized a based population cohort study to focus to the relationships between mode of delivery (cesarean section vs. vaginal birth) in the first pregnancy and the risk of subsequent preterm birth. With published data from ten retrospective cohort studies, for more than ten million women, our pooled analysis revealed that, compared with primary vaginal birth, cesarean section in the first pregnancy increased the risk of preterm birth (aRR 1.12, 95%CI 1.01–1.24) in subsequent pregnancies.

The uterine structure and/or intrauterine microenvironment may be changed by previous cesarean section [17]. These changes can elevate the risk of subsequent preterm birth in the next pregnancies. However, the pathogenesis of preterm birth in subsequent births of women who suffered a cesarean delivery remains unclear, but multiple hypotheses exist.

One possible risk factor that cervical trauma in the second stage of labor or unintentional incision into the uterine cervix during the previous cesarean section could disrupt the cervical integrity. This damage can affect the function of the cervix, and further increase the risk of preterm birth in future pregnancies. This phenomenon has been described in pregnant women by a Japanese obstetrician Koyama S [35]. Meanwhile, it was also found that compared to women with vaginal births, women with a full-term second-stage cesarean delivery have an increased risk of preterm birth in the subsequent pregnancy, as seen in a large retrospective cohort study [36].

Another possible explanation for the subsequent increase in preterm birth may be the formation of uterine scar after previous cesarean section. It has been found that adhesions created by the previous cesarean section could reduce utero-placental function and disturb the position of blastocyst implantation. These could further create sub-optimal conditions for fetal development [37]. A retrospective cohort study and a meta-analysis simultaneously reported that uterine scar dehiscence in a previous pregnancy was a potential risk factor for preterm delivery [38, 39]. Additionally, a large multi-high-income country study has also documented that an association was observed between preterm birth and decreasing clinician-initiated obstetric interventions, such as labor induction or cesarean delivery [40].

Alternatively, underlying reasons, such as higher body mass index, advanced maternal age, or other maternal medical characteristics (e.g., diabetes mellitus, preeclampsia, etc.), which are indications for the cesarean section in the first pregnancy can also be an important cause of preterm birth in the next pregnancies [41–44]. From a center database, the researchers found that gestational weight gain is independently associated with preterm birth in Peruvian pregnant women [41]. A study from the Swedish Medical Birth Register also found that advanced maternal age is associated with an increased risk of preterm birth irrespective of parity, especially very preterm birth [42]. In addition, previous observational studies have respectively reported that women with a history of preeclampsia and diabetes mellitus in a prior pregnancy were significantly associated with higher odds of preterm birth in American [43, 44].

In this study, our results indicate that previous cesarean section could increase the risk of preterm birth (before gestational week 37) in subsequent birth, but we showed that previous cesarean section was not associated with very preterm birth (before gestational week 32) in subsequent birth. Although other confounders may be related to very preterm birth which are not known or addressed here, an important explanation could be that too few articles (only four articles) included very preterm birth. After all, small individual studies might be underpowered and easy to cause false negative to identify the risk of results in the analysis.

Interpregnancy interval can affect the outcome of the subsequent pregnancy. A previous study reported that a short interpregnancy interval may increase the risk for abnormally invasive placenta in subsequent pregnancy [37], which may influence outcomes of next pregnancy. In this study, we also want to explore the relationship between interpregnnacy interval and preterm birth in subsequent. However, none of the included studies provided the time interval between a previous cesarean section and the subsequent conception. Future systematic reviews could compare the effects of different interpregnancy interval on preterm birth in subsequent.

Strength of our systematic review is the access to a relatively large sample size, including more than ten million women, and the consistent results of both before and after adjustments factors are provided in the preterm birth in subsequent pregnancies. However, some limitations of the study merit attention. Firstly, only developed cohort studies, including data from multicenter and national registries, were included in this meta-analysis, and the differences in the reporting of preterm birth rate could have affected the quality of the reported data. We would hope that future studies would also consider the previous mode of delivery and preterm birth in subsequent pregnancies in developing countries. Secondly, there was a high heterogeneity among the studies evaluating the risk of preterm birth in the combined analysis. However, Begg’s test and Egger’s test for each site-specific analysis showed no publication bias or small-study effects. Third, although including more than ten million women included, there are only ten published articles that are suitable to include in this review. The number of articles included seems too small. Fourth, while the RR-value reached statistical significance, it is should also note that the small size suggest that the clinical significance is not very strong.

In conclusion, this is the first systematic review and meta-analysis to our knowledge that showed that previous cesarean section could increase the risk of preterm birth in subsequent pregnancies. The result could provide policy makers, clinicians, and expectant parents to reduce the occurrence of unnecessary cesarean section.

## Supporting information

S1 Table PRISMA checklist. (DOC)

S1 Text Search strategy. (DOCX)
