# Supplementary material for: Mode of delivery and preterm birth in subsequent births: A systematic review and meta-analysis
Source: PLoS One. 2019 Mar 14;14(3):e0213784. doi: 10.1371/journal.pone.0213784 (PMC6417656; doi:10.1371/journal.pone.0213784)
Supplement: S1 Text — (DOCX) [file pone.0213784.s002.docx]

**S1 Search Strategy**

Search ((((("Delivery, Obstetric"[Mesh]) OR mode of delivery)) AND ((("Cesarean Section"[Mesh]) OR cesarean section) OR cesarean delivery)) AND (("Vacuum Extraction, Obstetrical"[Mesh]) OR vaginal delivery)) AND ((("Premature Birth"[Mesh]) OR preterm birth) OR preterm delivery) Filters: Publication date to 2018/11/27

*number of hits: 809*
